# Nonalcoholic Fatty Liver Disease as a Potential Risk Factor for Cardiovascular Disease in Patients with Type 2 Diabetes: A Prospective Cohort Study

**DOI:** 10.1155/2024/5328965

**Published:** 2024-06-26

**Authors:** Mohammad Dehghani Firouzabadi, Amirhossein Poopak, Ali Sheikhy, Fatemeh Dehghani Firouzabadi, Fatemeh Moosaie, Soghra Rabizadeh, Sara Momtazmanesh, Manouchehr Nakhjavani, Alireza Esteghamati

**Affiliations:** ^1^ Endocrinology and Metabolism Research Center (EMRC) Vali-Asr Hospital Tehran University of Medical Sciences, Tehran, Iran; ^2^ Department of Radiology and Imaging Sciences Clinical Center National Institutes of Health, Bethesda, USA

## Abstract

**Methods and Results:**

In this prospective cohort study, 1197 patients with type 2 diabetes (T2D) were divided into two groups (360 patients with NAFLD and 847 without NAFLD) and were followed for a median of 5 years for the incidence of CVD. Cox regression analysis was used to assess the association between NAFLD, liver enzyme level, aspartate aminotransferase to platelet ratio index (APRI), and the incidence risk of CVD and its subgroups (i.e., myocardial infarction, chronic heart disease, coronary artery bypass grafting, and percutaneous coronary intervention). There was a significant positive association between CVD incidence and NAFLD (HR = 1.488, 95% CI = 1.041–2.124, *p* value = 0.029). Although patients with NAFLD had higher levels of ALT and AST levels (*p* value = <0.001), there was no significant association between liver enzymes and the incidence risk of CVD when adjusted for different variables. Furthermore, NAFLD was associated with NAFLD APRI *Q* (2), APRI *Q* (3), and APRIQ (4) (1.365 (1.046–1.781), 1.623 (1.234–2.135), and 3.373 (2.509–4.536)), respectively.

**Conclusion:**

NAFLD increased the incidence risk of CVD in T2D. However, there was no association between liver enzymes (ALT, AST, ALK-P, and GGT) and a higher incidence risk of CVD in T2D when adjusted for confounding variables.

## 1. Introduction

Nonalcoholic fatty liver disease (NAFLD) is the most common chronic liver disorder in western countries [1]. The worldwide prevalence of NAFLD is estimated at 20% and is expected to be greater when it is in combination with obesity and diabetes [2, 3]. NAFLD spectrum ranges from simple steatosis to nonalcoholic steatohepatitis [4].

Although the etiology of NAFLD is unknown, insulin resistance is a key mechanism of lipid deposition in hepatocytes leading to steatosis and potentially steatohepatitis in patients with diabetes [4–6]. Obesity and T2D that are associated with peripheral insulin resistance [7, 8] result in the secretion of multiple secreted factors, that is, free fatty acids, tumor necrosis factor-alpha, interleukin-6, and other proinflammatory cytokines leading to acceleration of steatosis and increasing oxidative stress, inflammation, dyslipidemia, visceral fat, low adiponectin, distribution of ectopic adipose tissue, endothelial dysfunction, and postprandial dyslipidemia that are the main factors that lead to and further aggravate the course of NAFLD, as well as accelerating the progression of atherosclerosis and development of cardiovascular diseases (CVD) [9–12] which is the most common cause of mortality in NAFLD patients [13–15]. For example, Targher et al. in a cross-sectional study demonstrated that NAFLD is associated with a higher prevalence of CVD, independent of classical risk factors, and 69% of 2839 patients with diabetes had NAFLD [16]. According to a wide range of current evidence, the role of NAFLD in the incidence risk of CVD is not yet clear. A group of studies showed that there was an association between NAFLD and CVD risk in patients with diabetes [16–19], while other studies could not approve this association [20, 21]. A possible explanation for this heterogeneity in the previous studies could be due to small sample size, the different methods used for the detection of NAFLD (e.g., ultrasound, computed tomography, and liver biopsy), the difference in the study participants and the duration of the study and follow-up periods. Therefore, it is necessary to assess the possible role of NAFLD as a new cardiometabolic risk factor for long-term CVD outcomes, regardless of association with traditional risk factors, in patients with diabetes.

This prospective cohort study aimed to investigate the association of NAFLD and liver enzymes (AST, ALT, ALK-P, and GGT) with the incidence of CVD in patients with T2D in a median of 5-year follow-up [22, 23].

## 2. Methods

### 2.1. Study Population

In this prospective study, we enrolled 1197 patients with a history of T2D who attended Vali-Asr Hospital, which was affiliated with Tehran University of Medical Sciences, between February 1st, 2010, and December 31st, 2020. The baseline characteristics of the patients (cholesterol levels and other lipid and glycemic indices) were measured and 637 patients with diabetes who did not have fatty liver and 560 patients with diabetes who had fatty liver were followed for a median of 5 years. A single-blinded radiologist performed an abdominal ultrasound examination of the liver and biliary tree (Hitachi EUB 405 apparatus equipped with a convex × 3.5 MHz probe, intra- and interobserver coefficients of variation <5%) to diagnose NAFLD. NAFLD was diagnosed with the definite presence of grade 3 hepatic steatosis defined based on hyperechogenicity relative to the renal parenchyma, reductions in the ultrasound beam, and/or the visualization of the diaphragm and intrahepatic vessels/structures reduced or absent, consistence evidence of severe hepatic steatosis with or without focal fatty sparing [24, 25]. We excluded patients having or susceptible to other type of hepatitis and fatty liver (with a history of autoimmune hepatitis, alcohol abuse, viral hepatitis, hemochromatosis, Wilson's disease, primary biliary cirrhosis, use of drugs inducing hepatotoxicity, and/or rapid weight loss), and patients without fatty liver who were diagnosed with fatty liver during our follow-up, so they were removed under physical examination and laboratory investigations (i.e., iron studies, ceruloplasmin, antinuclear and antismooth muscle antibody, and urinary copper). We also regularly recorded the status of alcohol consumption and possible chronic causes of non-NAFLD in each participant. Participants with a history of kidney disease (creatinine (Cr) > 2 mg/dl or estimated glomerular filtration rate (eGFR) < 30 cc/min), familial hypercholesterolemia, liver dysfunction, epilepsy hypothyroidism, and hemoglobinopathy were excluded. Furthermore, women who were pregnant or taking oral contraceptives or hormone replacement therapy were excluded. Moreover, we excluded any patient who drinks any amount of alcohol. Patients with other types of diabetes and metabolic conditions (type 1 diabetes, insulin-requiring type 2 diabetes, gestational diabetes, secondary diabetes to malignancies, pancreatitis, and other metabolic conditions) were also excluded. After obtaining a written consent form, a total of 637 diabetic patients without fatty liver and 560 diabetic patients with fatty liver were selected. All participants underwent a physical examination and laboratory investigations at the start of the study. Informed consent was obtained from all individuals to participate in the study.

### 2.2. Examination

The weight and height of the included patients were measured in light clothing and no shoes. By dividing weight in kilograms by square of height in meters, the body mass index (BMI) was calculated. The middle point between the iliac crest and the rib cage, while the patient was standing relaxed, was chosen to measure the waist circumference using a nonstretchable measuring tape. Systolic blood pressure (SBP) and diastolic blood pressure (DBP) were measured three times at a 5-minute interval after 10 minutes of seated rests using Omron M7 digital sphyg142 manometers (Hoofddorp, The Netherlands) calibrated with appropriately sized cuffs that covered at least 80% of the right arm of the subjects. The mean values of second and third times were documented for further analysis. Hypertension was determined with SBP greater than 140 mmHg, or DBP greater than 90 mmHg, or a previous diagnosis of hypertension and current treatment with antihypertensive drugs. Demographic information, current smoking, and medication use status were self-reported during the interview.

### 2.3. Laboratory Investigations

For laboratory evaluations, 10 ml of venous blood was drawn from each patient after 12–14 h of fasting overnight. The samples were kept at a temperature of 4 to 8 C until they were sent to the appropriate calibrating laboratories and centrifuged (1500 rmp, for 10 min, at a standard room temperature of 21 C). At a stored temperature of −70°C, laboratory evaluations were carried out on the extracted serum stored. High-performance liquid chromatography (A1C, DS5 Pink kit; Drew, Marseille, France) was used to investigate glycated hemoglobin (HbA1c). Enzymatic calorimetric methods using the glucose oxidase test were used to measure fasting blood sugar (FBS) and glucose two hours after prandial (2hpp). Total cholesterol, high-density lipoprotein cholesterol (HDL-c), low-density lipoprotein cholesterol (LDL-c), and triglycerides were measured using enzyme methods. The central reference laboratory (Tehran, Iran) approved the kits used in this study [26]. Serum creatinine levels were evaluated using the Jaffe method (Parsazmun, Karaj, Iran). To calculate the estimated glomerular filtration rate (eGFR), the formula for modification of diet in renal disease (MDRD) was used (eGFR = 186 × (serum creatinine) − 1.154 × (age) − 0.203 × (0.742 if female)) [27]. The APRI score was calculated using ((AST/upper limit of the normal AST range) × 100)/Platelet count formula. Serum levels of alanine aminotransferase (ALT), aspartate aminotransferase (AST), alkaline phosphatase (ALK-P), and glutamyl transferase (GGT) were analyzed using enzymophotometry. The IFCC (International Federation of Clinical Chemistry and Laboratory Medicine) method (ALT intraassay CV = 3.7%, AST intraassayCV = 2.5%, and GGT intraassay CV = 2.2%) was used to measure serum ALT, AST, and GGT, and the DGKC (Deutsche Gesellschaft für KlinischeChemie) method (intraassay CV = 1.5%) was used to measure serum ALK-P [28, 29]. According to standardized criteria, the ALT level >30 IU/L in women and >40 IU/L in men, the AST level >30 IU/L in women and >36 IU/L in men, ALK-P levels >306 U/L in both genders, and GGT levels >40 IU/L in women and >60 U/L for men are considered elevated. These tests were performed using commercial Parsazmunkits (Tehran, Iran) and a Hitachi 704 automatic analyzer (Tokyo, Japan). For the diagnosis of viral hepatitis (hepatitis B and C), commercially available enzyme-linked immunosorbent assay kits (DRG Diagnostics GmbH, Germany) were used to measure the hepatitis B antibody, hepatitis B surface antigen, hepatitis B surface antibody, hepatitis B core antibody, hepatitis B antigen, and hepatitis C antibodies. All laboratory tests were assayed and attested by investigators and technicians blinded to the status of the patients.

### 2.4. CVD Assessment

Cardiovascular disease (CVD) was stated if the patient had nonfatal coronary artery disease diagnosed by a physician, positive findings on noninvasive tests (i.e., myocardial perfusion scan, exercise tolerance test, transthoracic echocardiography, and multidetector computed tomography coronary angiography) that promptly needed initiation of antiischemic treatment. Angina pectoris, coronary insufficiency, acute coronary syndrome (ACS), myocardial infarction (MI), coronary artery bypass graft surgery (CABG), coronary angioplasty, percutaneous coronary intervention, and other revascularization procedures were considered nonfatal coronary artery disease.

In the result, revascularization is defined as the need for a PCI or CABG procedure. MI/ACS defined as myocardial infarction is treated with pharmaceutical agents, without any invasive procedure. Congestive heart failure (CHF) is diagnosed according to the Framingham Diagnostic criteria.

### 2.5. Statistical Analysis

Version 25 of the SPSS software and the R programming language (V 4.2.2) were used to perform statistical analysis of the recorded data. To test for the normality of the study population, Kolmogorov–Smirnov and Shapiro–Wilk normality tests and *P*–*P* plot and histograms were used. A univariate analysis of potential continuous and categorical risk factors was performed using the *t*-test and the chi-square test, respectively. Data are reported as mean ± standard deviation (SD) for normal distributed continuous variables, median with 95% CI for skewed variables, and as proportions for categorical variables. Multivariate Cox regression analysis was performed to assess the association between NAFLD and liver enzymes (i.e., AST, ALT, ALK-P, and GGT) and the incidence of CVD and its subtypes (i.e., MI, PCI, CABG, and CHF), by addressing potential confounders including, duration of diabetes, diabetes control (2hpp), hypertension, dyslipidemia, and CKD. We created two different models. In the first model, CVD is considered as the outcome and in the second model CVD subtypes are considered the outcomes. Regarding CVD, test exposures were adjusted for age, sex, duration of diabetes, 2hpp, FBS, HDL, LDL, creatinine, and BMI separately. As for CVD subtypes, adjustment was performed for age, sex, duration of diabetes, and FBS. The adjusted variables were selected on the significance in the univariate model. All variables with a *P* value less than 0.1 entered the model. The level of statistical significance was set to be a *P* value < 0.05.

## 3. Results

### 3.1. Baseline Characteristics of the Study Population

**Figure 1:**
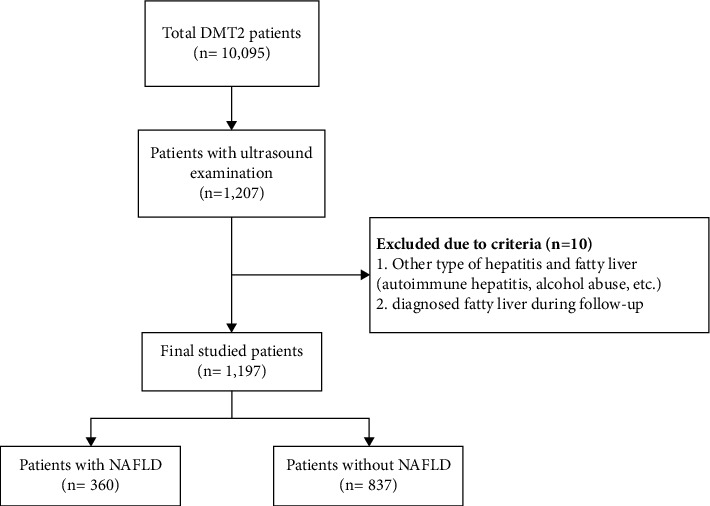
Flowchart of studied patients.

In this study, 1197 patients (43.5% male) who met our inclusion criteria were enrolled and followed for a median of 5 years (min = 1 year, max = 40 years). Patients with NAFLD (360 patients with a mean age of 53.19 ± 10.36) had significantly lower age, duration of diabetes, duration of use of oral antidiabetic drugs, and creatinine compared to patients without NAFLD (837 patients with a mean age of 57.38 ± 11.31) (Figure 1). However, patients with NAFLD had a higher DBP, a serum level of total cholesterol, uric acid, AST, and ALT, and also, their BMI was higher than that of patients without NAFLD. There were no significant differences in sex, SBP, FBS, 2hpp, HbA1c, HDL-c, LDL-c, triglycerides, ALK-P, and GGT between the two groups (Table 1).

### 3.2. NAFLD and Liver Enzymes and Incidence of CVD

**Figure 2:**
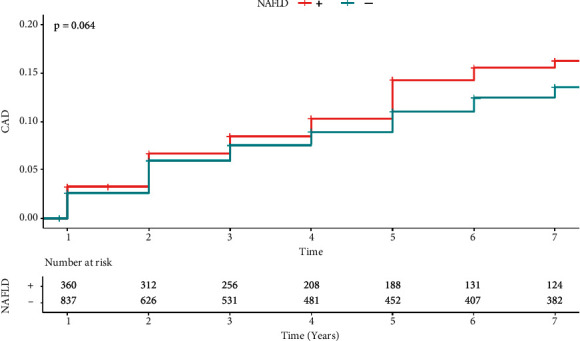
The Kaplan–Meier CAD incidence analysis of patients with and without NAFLD.

NAFLD was not associated with CVD incidence in the univariate model (Figure 2) (*P*=0.064); hence, there was a significant positive association between NAFLD and CVD incidence after adjustment for confounder variables (aHR = 1.4887 (1.041–2.124), *P*=0.029).

There was no statistically significant association between liver enzymes and CVD incidence (Table 2).

### 3.3. NAFLD and Liver Enzymes and Incidence of CVD Subgroups

Among 1207 patients, 12, 62, 23, and 128 patients experienced congestive heart failure (CHF), coronary artery bypass grafting (CABG), myocardial infarction (MI), and percutaneous coronary intervention (PCI), respectively. There were no significant associations between NAFLD and liver enzymes with the risk of CVD incidence of subgroups, except for ALT levels and MI incidence (HR: 1.01 [1.01, 1.02], *P*=0.005) and ALK-P levels with CHF (HR: 1.02 [1.01, 1.04], *P*=0.012) (Table 2).

#### 3.3.1. Association of APRI and NAFLD

The relationship between APRI and NAFLD is summarized in Table 3. After controlling for confounding variables, the values of APRI *Q* (2), APRI *Q* (3), and APRI *Q* (4) were considerably higher in patients with NAFLD (OR: 1.365 (1.046–1.781), 1.623 (1.234–2.135), and 3.373 (2.509–4.536)), respectively. The duration of diabetes, fasting blood sugar level, sex, age, 2hpp, creatinine, and BMI were corrected using a multivariate-adjusted tertiary model.

## 4. Discussion

In this longitudinal study, we found that patients with T2D who had NAFLD had higher CVDs, and there was a strong association between NAFLD and the incidence risk of CVD in the diabetic population when adjusted for different variables in the adjusted tertiary multivariate model. There was no association between liver enzymes (ALT, AST, ALK-P, and GGT) and a higher incidence risk of CVD in T2D. In addition, there was no relationship between AST, ALT, and GGT enzymes with the incidence risk of CVD subgroups such as PCI, MI, CABG, and CHF.

Previous studies assessed the association between NAFLD and CVD incidence in the general or diabetic population by diagnosis of NAFLD based on serum liver enzymes and fatty liver index, liver ultrasound, or liver biopsy.

Studies using liver biopsy to detect NAFLD showed that the most common cause of death was CVD in patients with evidence of liver fibrosis on their histopathology [30–32]. Furthermore, another study reported that a higher mortality rate was found in the fibrosis stage of 3-4 [33]. In addition, a study determined that there was a significant relationship between apparent steatohepatitis at biopsy and cardiac diseases such as left atrial enlargement and increased left ventricular mass [34]. The results of our study support these findings.

A group of different studies, in contrast to our results, determined that there was a strong association between the elevated level of *γ*-glutamyl transferase (GGT) and the presence and progression of CVD and severe cardiovascular outcomes leading to death [35, 36]. Furthermore, there were some reports on the association between an increase in the level of alanine aminotransferase (ALT) and a higher 10-year CVD risk after adjustment for metabolic risk factors and alcohol use [37, 38]. A possible explanation for the different results in our study could be a shorter follow-up period compared to previous studies.

Liver ultrasound could detect patients with a high incidence risk of CVD based on previous studies that showed a strong association between ultrasound severity of steatosis and coronary/carotid atherosclerotic disease [39, 40].

APRI was found to have a significant relationship with NAFLD in this study. A recent systematic review found that the APRI risk stratifies morbidity and death in individuals with NAFLD, which is consistent with our findings [41]. APRI can also detect fibrosis in NAFLD, according to a recent cross-sectional study in Iran [42]. In addition, in patients with NAFLD, a retrospective cohort research in Canada compared the predictive efficacy of noninvasive diagnostic procedures such as APRI with liver histology and liver venous pressure gradient (HVPG). Their findings revealed that APRI can predict patient outcomes for NAFLD and that it can be used to monitor, stratify risk, and identify targeted therapies [43].

### 4.1. Pathophysiology of NAFLD and CVD

The first possible explanation for CVD incidence in NAFLD is insulin resistance and abdominal obesity in this group of patients. Elevated insulin level leads to a change in the cellular free fatty acid (FFA) storage pathway, resulting in increased storage of the fatty acids in the skeletal muscle and liver rather than adipose tissue. This alternation in the FFA pathway leads to fat accumulation in liver cells causing insulin resistance resulting in increased oxidative stress in hepatocytes that causes abnormal adipocytokine profile and endothelial dysfunction and activation of further cardiometabolic dysfunctional cascade [41, 44]. In addition, some studies showed that elevated FFA and very low-density lipoproteins had a role in producing more plasminogen activator inhibitor 1 leading to endothelial dysfunction and stimulation of atherosclerosis resulting in adverse cardiovascular events [42, 43].

The second possible mechanism could be increased inflammatory mediators due to liver dysfunction and chronic disease. Furthermore, according to a group of studies, patients with NAFLD had a higher level of inflammatory mediators and prothrombotic, including high-sensitive C-reactive protein, tumor necrosis factor *α*, and interleukin 6, fibrinogen, and plasminogen activator inhibitor that stimulated the nuclear factor-*κ*B (NF-*κ*B) and c-Jun N-terminal kinase (JNK) pathway [45, 46]. The increase in nuclear factor-*κ*B (NF-*κ*B) plays a role in the activation of more genes in hepatocytes (e.g., intercellular adhesion molecule 1 and monocyte chemoattractant protein 1) related to the production of proinflammatory and atherogenic factors and the accumulation of systemic inflammatory mediators [47]. Furthermore, excess c-Jun N-terminal kinase (JNK) can lead to insulin resistance through its effect on the intracellular signaling pathway causing deactivation of insulin receptor [48]. However, some studies determined that there was no association between endothelial dysfunction and NAFLD that may be related to considering a mild form of NAFLD, as the other studies revealed more cardiovascular events and endothelial dysfunction in the severe form of NAFLD [49, 50]. Furthermore, there is some evidence that showed larger intima media thickness and more calcified and noncalcified coronary plaques in patients with NAFLD, explaining the higher incidence and prevalence of CVD in this group compared to patients without NAFLD [51, 52].

### 4.2. The Role of NAFLD in CVD Subtypes

Based on clinical studies, NAFLD plays an important role in the presence and progression of different manifestations of CV, such as left ventricular dysfunction, atherosclerotic CV disease, coronary heart disease [53] and structural cardiac abnormalities (valve dysfunction, myocardial hypertrophy, heart failure), arrhythmias (atrial fibrillation, premature ventricular beats, and nonsustained ventricular tachycardia [35]), and cerebrovascular and thromboembolic events (ischemic and hemorrhagic stroke) [54, 55], suggesting that its contribution may be independent of the presence of traditional CV risk factors [56–58].

### 4.3. Treatment of NAFLD

Some strategies are recommended to avoid further adverse cardiovascular risk in patients with NAFLD such as lifestyle changes (e.g., an appropriate and healthy diet such as Mediterranean diet [59], regular physical activity, and cessation of smoking), pharmacotherapy to control components of metabolic syndrome such as antidiabetic drugs (e.g., pioglitazone [60], glucagon-like peptide-1 receptor agonists [61], and dipeptidyl peptidase-4 inhibitors [62]), antihypertensive drugs (e.g., angiotensin receptor blockers [63]), and lipid-lowering agents (e.g., statins [64, 65] and omega-3 polyunsaturated fatty acids [66]), and pharmacotherapy to control liver disease in patients with severe NAFLD to avoid cirrhosis and its consequences. Some treatments such as orlistat, vitamin *E* [67], and bariatric surgery [68] are recommended to give some treatments such as orlistat, vitamin *E* [67], and bariatric surgery [68] to manage NAFLD in patients with current cardiometabolic disease based on a group of studies.

Moreover, recent studies revealed that antianginal agent ranolazine considerably reduced serum ALT and AST activities in patients with stable CAD and NAFLD [69].

This study had several strengths. First, to our knowledge, it is the first prospective longitudinal study that evaluated the association between NAFLD and liver enzymes (AST, ALT, ALK-P, and GGT) with the incidence risk of CVD complications in patients with T2D, and the prospective design of this study can establish a causal relationship between the measured variables. Second, previous studies showed the association between NAFLD and CVD prevalence in the general population; however, only a few have considered its possible role as a risk factor and a contributor to the development of CVD complications in patients with diabetes. Third, the adequate sample size and the exclusion of other liver diseases have increased the representativeness of our results. However, this study also had some limitations. We considered NAFLD in the present study according to the definite signs of liver steatosis (grade 1–3 liver steatosis on abdominal ultrasound), our results may not be applied to patients with earlier stages of liver steatosis on ultrasound or those individuals with ultrasound-detectable NAFLD. Further long-term, prospective observational studies on various ethnic groups at all stages of NAFLD are recommended to determine the possible role of NAFLD as a predictive factor for the development of CVD complications of T2D. Moreover, we did not evaluate other potential confounders including patient's medication and physical activity in this study.

## 5. Conclusion

In the present study, although after adjustment for different variables, only NAFLD increased the incidence risk of CVD in T2D after 5 years, there was a positive association between NAFLD and liver enzymes (ALT, AST, ALK-P, and GGT) in increasing the incidence risk of CVD in T2D. NAFLD patients with simultaneous T2DM can be acknowledged as special risk groups, which have the highest risk for adverse cardiovascular events and mortality. Diagnosis of NAFLD is crucial in patients with diabetes, and it is beneficial to evaluate subclinical atherosclerosis. Currently, there are no specific approved treatments for NAFLD, so applying the Mediterranean diet is the best option for both prevention and treatment in these patients.

## Figures and Tables

**Table 1:** Baseline characteristics of the study population based on the incidence of NAFLD.

		With incident NAFLD	Without incident NAFLD	*P* value
Sex	Female	205	475	0.651
	Male	155	362	
Age (year)		53.2 ± 10.4	57.4 ± 11.3	<0.001
Duration of diabetes (year)		5.9 ± 5.6	8.0 ± 7.8	<0.001
SBP (mmHg)		128.5 ± 15.0	135.6 ± 75.9	0.082
DBP (mmHg)		80.5 ± 7.2	78.8 ± 7.8	<0.001
Drug duration (year)		2.5 ± 1.0	2.8 ± 2.5	<0.002
FBS (mg/dL)		153.8 ± 55.0	155.6 ± 51.3	0.590
2hpp (mg/dL)		214.9 ± 92.7	216.1 ± 82.6	0.824
HbA1C (%)		7.4 ± 1.5	7.9 ± 6.0	0.187
Total cholesterol (mg/dL)		184.0 ± 42.0	177.4 ± 43.1	0.015
HDL-c (mg/dL)		44.7 ± 12.9	45.1 ± 12.3	0.612
LDL-c (mg/dL)		103.4 ± 33.6	99.8 ± 34.3	0.092
TG (mg/dL)		184.2 ± 97.0	173.3 ± 102.5	0.089
Cr (mg/dL)		0.97 ± 0.19	1.01 ± 0.26	0.009
eGFR		76.5 ± 16.7	73.5 ± 18.5	0.008
Uric acid level (mg/dL)		5.4 ± 1.4	5.1 ± 1.3	0.003
BMI (kg/m^2^)		30.9 ± 6.0	29.3 ± 5.3	<0.001
AST (IU/L)		29.0 ± 18.4	23.8 ± 17.4	<0.001
ALT (IU/L)		39.9 ± 27.4	29.0 ± 22.1	<0.001
ALK-p (IU/L)		177.1 ± 83.8	172.2 ± 92.2	0.444
GGT (IU/L)		43.1 ± 56.1	42.2 ± 101.2	0.907
CVD (%)		67 (18.82%)	175 (20.80%)	0.434
MI		6 (1.67%)	17 (2.0%)	0.312
Revascularization		57 (15.83%)	157 (18.53%)	0.089
CHF		2 (0.56%)	2 (0.24%)	0.812

SBP, systolic blood pressure; DBP, diastolic blood pressure; FBS, fasting blood sugar; 2hpp, two-hour postprandial glucose; HbA1C, hemoglobin A1C; HDL, high-density lipoproteins; LDL, low-density lipoproteins; TG, triglycerides, Cr, creatinine; UA, uric acid; BMI, body mass index; ALT, alanine aminotransferase; AST, aspartate aminotransferase; ALK-P, alkaline phosphatase; GGT, glutamyl transferase; eGFR, estimated glomerular filtration rate; NAFLD, nonalcoholic fatty liver disease; MI, myocardial infarction; CHF, congestive heart failure.

**Table 2:** Association between incidence of CVD subgroups with NAFLD and liver enzymes.

		AST	ALT	ALK-P	GGT	NAFLD
MI	Yes	28.41 ± 27.43	27 [16, 36]	172.63 ± 85.62	20 [15, 40]	6
	No	24.44 ± 18.38	27 [16, 36]	173.04 ± 85.62	24 [22, 28]	217
	*P* value	0.327	0.733^*∗*^	0.985	0.426^*∗*^	0.607
	HR	1.00 [0.99, 1.02]	1.01 [1.01, 1.02]	1.00 [0.99, 1.01]	0.99 [0.97, 1.02]	1.46 [0.59, 3.65]
	*P* value	0.732	0.005	0.961	0.451	0.416

Revascularization	Yes	24.12 ± 18.21	28.31 ± 12.12	164.23 ± 61.23	33.31 ± 20.65	57
	No	24.51 ± 18.23	30.28 ± 18.92	173.21 ± 78.64	42.58 ± 15.51	305
	*P* value	0.338	0.341	0.531	0.772	0.144
	HR	0.99 [0.98, 1.02]	1.00 [0.98, 1.01]	1.00 [0.99, 1.01]	1.00 [0.99, 1.01]	1.20 [0.65, 2.24]
	*P* value	0.881	0.722	0.659	0.774	0.561

CHF	Yes	18.21 ± 7.21	36.00 ± 5.62	237.21 ± 24.12	40 [20, 50]	2
	No	28.41 ± 8.21	30.59 ± 23.45	172.60 ± 89.42	24 [23.28]	312
	*P* value	0.291	0.712	0.036	0.922^*∗*^	0.357
	HR	1.01 [1.00, 1.01]	1.01 [1.00, 1.01]	1.02 [1.01, 1.04]	0.99 [0.98, 1.00]	2.12 [0.94, 4.12]
	*P* value	0.912	0.212	0.012	0.843	0.081

ALT, alanine aminotransferase; AST, aspartate aminotransferase; ALK-P, alkaline phosphatase; GGT, glutamyl transferase; NAFLD, nonalcoholic fatty liver disease; MI, myocardial infarction; CHF, chronic heart disease; CABG, coronary artery bypass grafting; PCI, percutaneous coronary intervention. ^*∗*^Mann–Whitney *U* test.

**Table 3:** Association between incidence of NAFLD and APRI.

	NAFLD		
	Risk estimate	95% CI	*P* value
APRI_Q (2)	1.365	1.046–1.781	0.022
APRI_Q (3)	1.623	1.234–2.135	0.001
APRI_Q (4)	3.373	2.509–4.536	<0.001

Data were adjusted for age, sex, duration of diabetes, fasting blood sugar, and 2hpp in the tertiary multivariate-adjusted model. APRI, aspartate aminotransferase to platelet ratio index. The reference category is APRI Q1.

## Data Availability

All the data generated or analyzed during this study are included within this submitted article.
